# Prognostic Significance of Bone Marrow Computed Tomography Attenuation in Patients With Non-small Cell Lung Cancer Without Curative Surgery

**DOI:** 10.7759/cureus.102922

**Published:** 2026-02-03

**Authors:** Shiro Ishii, Hiroki Suenaga, Ryo Yamakuni, Yasuhiro Abe, Yoshiki Endo, Shigeyasu Sugawara, Daichi Kuroiwa, Hirofumi Sekino, Kenji Fukushima, Hiroshi Ito

**Affiliations:** 1 Department of Radiology and Nuclear Medicine, Fukushima Medical University, Fukushima, JPN

**Keywords:** bone marrow, ct number, fdg, non-small cell lung carcinoma (nsclc), overall survival (os)

## Abstract

Introduction

Bone marrow fluorodeoxyglucose (FDG) uptake and bone marrow computed tomography (CT) attenuation are imaging biomarkers reflecting bone marrow activity. This study aimed to investigate whether bone marrow CT attenuation is associated with the prognosis of patients with non-small cell lung cancer (NSCLC).

Methods

Overall, 160 patients with NSCLC who underwent fluorodeoxyglucose positron emission tomography (FDG-PET)/CT staging between January 2018 and December 2019 were retrospectively reviewed. Using an elliptical region of interest in an axial image of the fused PET and CT scans, the mean standardized uptake value of 18F-FDG was measured for the vertebral bone marrow and liver as reference areas, and the mean CT attenuation value of the femoral bone marrow. Survival curves were constructed using the Kaplan-Meier method to calculate overall survival (OS) rates. The predictive value of the variables for OS was evaluated using the log-rank test in univariate analysis and the Cox proportional hazards regression model in multivariate analysis. Age, sex, stage, serum albumin level, C-reactive protein (CRP) level, neutrophil-to-lymphocyte ratio (NLR), platelet-to-lymphocyte ratio (PLR), bone marrow-to-liver ratio (BLR), and bone marrow CT number were evaluated in the OS analysis. All continuous variables in the survival analysis were dichotomized according to the cut-off values determined by receiver operating characteristic curve analysis.

Results

Of 160 patients, 105 underwent surgery; 24 (22.9%) experienced recurrence, and 22 (21.0%) died during the 60-month clinical follow-up. Of the 55 patients who did not undergo surgery, 37 (67.2%) died during follow-up. Bone marrow CT number showed a weak correlation with BLR (γ=0.257) and BMI (γ=0.323). In the analysis of patients who received curative surgery for NSCLC, the following were significantly associated with prognosis in the univariate analysis: age (P<0.001), tumor-node-metastasis stage (P<0.001), albumin (P=0.029), serum CRP level (P=0.020), NLR (P=0.013), and PLR (P=0.001); however, bone marrow CT number (P=0.233) was not. In the analysis of patients who did not undergo curative surgery, only the bone marrow CT number was significantly associated with prognosis in the univariate analysis (P=0.018) but not in the multivariate analysis (P=0.123), indicating that bone marrow CT attenuation was not an independent prognostic factor.

Conclusion

This study suggests that lower bone marrow CT attenuation is associated with better survival in patients with non-small cell lung cancer who did not undergo curative surgery in univariate analysis. However, as this association did not remain significant after multivariate adjustment, bone marrow CT attenuation should be regarded as an exploratory or adjunctive prognostic imaging marker rather than an independent predictor.

## Introduction

Lung cancer is the most common cancer worldwide and one of the most fatal malignant tumors [1, 2]. Non-small cell lung cancer (NSCLC) is a common type of lung cancer, and various prognostic factors have been reported, including tumor-node-metastasis (TNM) classification and clinical, biochemical, and hematological factors. Systemic inflammation is significantly associated with the prognosis of various types of cancer. C-reactive protein (CRP) in the serum and the neutrophil-to-lymphocyte ratio (NLR) and platelet-to-lymphocyte ratio (PLR), calculated by dividing the neutrophil and platelet count, respectively, by the lymphocyte count, are known serum inflammatory markers reflecting systemic inflammatory responses. They have been reported to reflect systemic inflammatory responses in various conditions, including malignant diseases, and predict the prognosis of malignant tumors such as lung cancer [3-6].

Positron emission tomography (PET) using F-18 fluorodeoxyglucose (18F-FDG) is a widely accepted diagnostic imaging method for the evaluation of various neoplastic and inflammatory diseases. In patients with malignant tumors, bone marrow uptake of FDG and the bone marrow-to-liver ratio (BLR) have been reported to reflect bone marrow activation in response to inflammatory states, and their prognostic value has been reported in various cancers [7, 8]. Lee et al. reported that increased bone marrow FDG metabolism is a strong prognostic factor for NSCLC [9, 10]. Markers for predicting the prognosis of lung cancer are useful for determining treatment strategies and risk stratification, providing information to patients, and monitoring treatment efficacy. By comprehensively evaluating these prognostic markers, it may be possible to develop individualized treatment strategies.

The CT number obtained from CT scans reflects tissue composition and is typically expressed in Hounsfield units (HU). CT number measurement can help estimate the presence of fat, calcification, and hematoma. The bone marrow CT number (BM-CT number) obtained from the femoral bone marrow increases in cases of anemia, various blood disorders, inflammation, and the administration of G-CSF agents [11-13]. These studies reported that the BM-CT number and 18F-FDG values of the bone marrow are well correlated, suggesting that the CT values of the bone marrow may reflect the prognosis of patients with lung cancer. While FDG uptake reflects metabolically active bone marrow and systemic inflammatory status, BM-CT number may capture distinct structural changes such as marrow fat composition, which are not directly assessed by PET-based metrics. The prognostic relevance of these CT-derived features in non-small cell lung cancer remains insufficiently explored.

Additionally, CT has the advantage of being more widely available than FDG-PET and may serve as a simpler prognostic indicator than bone marrow FDG uptake. Therefore, this study aimed to investigate the effect of the number of BM-CT on the prognosis of NSCLC.

## Materials and methods

Patients

The electronic medical records of patients with lung cancer who underwent FDG-PET/CT staging between January 2018 and December 2019 were retrospectively reviewed. Patients whose blood data, including hemoglobin, white blood cell (WBC) count, platelet (Plt) count, and C-reactive protein, were obtained within 14 days of PET/CT examination were enrolled. Patients who had small cell lung cancer, a history of another malignancy, received any treatment for lung cancer before FDG-PET/CT examination, or been lost to clinical follow-up within 12 months after FDG-PET examination were excluded from the study. Finally, 160 patients with NSCLC were enrolled. After staging the NSCLC workup, all patients were scheduled for drug therapy, including chemotherapy, molecular targeted drug therapy, and immune checkpoint therapy; radiation; and surgery, including lobectomy, bilobectomy, or pneumonectomy with systematic lymph node dissection, according to their stage and clinical condition.

The histopathological and clinical stages of the patients were assessed according to the eighth edition of the cancer staging guidelines of the Japan Lung Cancer Society.This staging system is consistent with the 8th edition of the TNM classification proposed by the International Association for the Study of Lung Cancer (IASLC) [14]. Patients diagnosed with progressive disease or newly developed metastatic lesions received further palliative treatment, including drug and radiation therapies, depending on their clinical condition and site of recurrence. The patients were followed up for up to 60 months.

Patient characteristics, including age, sex, surgery, history of medication therapy and radiotherapy, hemoglobin, and blood cell counts, including neutrophils, platelets, lymphocytes, LDH (lactate dehydrogenase), and CRP, were extracted from the patients’ medical records. The NLR was calculated by dividing the absolute neutrophil count by the absolute lymphocyte count. The PLR was defined as the absolute platelet count divided by the absolute lymphocyte count.

PET/CT protocol

Fluorine-18 fluorodeoxyglucose positron emission tomography/computed tomography (18F-FDG-PET/CT) data were acquired using a 128-slice CT with Biograph mCT (Siemens Healthineers, Erlangen, Germany). All patients fasted for at least 4 h or skipped meals before their examination. They were each injected with 4MBq/kg FDG, approximately 1 h after which the PET/CT examination was started. The PET data were acquired from the upper thigh to the head during shallow breathing. The acquisition time was 2-3 min per bed position, with six to eight bed positions (each 21.8 cm). A 200 × 200 matrix was used for the analysis. PET data were reconstructed using ordered subset expectation maximization, containing three iterations and 21 subsets with time-of-flight, point spread function, and a Gaussian filter of 3 mm full-width at half-maximum. Attenuation correction was performed based on the CT data derived from the CT scan acquired before the PET scan. Acquisition parameters for CT were as follows: tube voltage, 120 kVp; auto mAs (reference tube current, 80 mAs); rotation time, 0.5 s; matrix, 512 × 512; and reconstruction using a 3-mm slice thickness and 2-mm increments. CT data were acquired during expiratory breath holding.

Measurement

The mean standardized uptake value (SUV) of 18F-FDG for the liver and vertebral bone marrow, and the mean CT attenuation value of the femoral bone marrow were measured using an elliptical region of interest (ROI) in an axial image of fused PET and CT images. An elliptical area about 60 mm in diameter was placed on the middle part of the right lobe, confirming the absence of background pathological lesions such as tumors or cysts. The ROI of the vertebra was placed at the center of Th11, Th12, and L3-L5 using a sagittal CT scout tool. Bone marrow SUV was averaged from the values obtained at Th11, Th12, L3, L4, and L5. The bone marrow/liver ratio (B/L ratio) was calculated by dividing the bone marrow SUV by the liver SUV to assess bone marrow FDG uptake. The ROI of the femoral BM was placed on the axial CT images at the level immediately distal to the lesser trochanter, where trabecular bone is minimal and not visually apparent. An elliptical ROI was manually drawn within the medullary cavity, carefully avoiding cortical bone and any visible trabecular structures. The ROI size was adjusted according to individual anatomy to maximize inclusion of homogeneous marrow while excluding bone cortex [11, 12].

**Figure 1:**
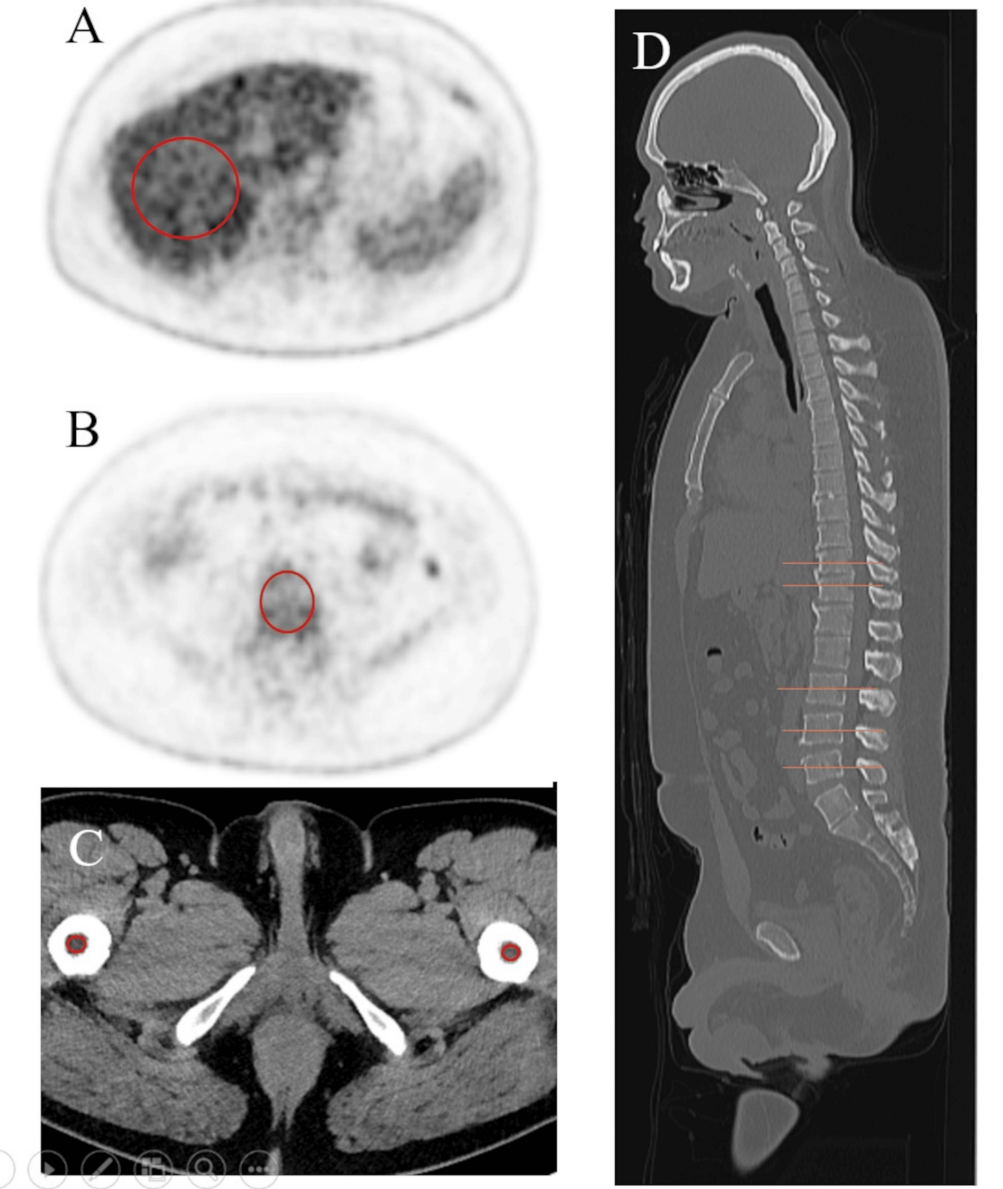
Placement of regions of interest (ROI). The ROIs were placed at the center of the Th11, Th12, L3, L4, and L5 vertebrae (B), using the scout tool (D), and the obtained mean standardized uptake values (SUVs) were averaged (Vertebral-SUV). The elliptical ROI of the liver was placed on the middle part of the right lobe (A), and the mean SUV was measured (Liver-SUV). Bone marrow-to-liver ratio (BLR) was calculated by dividing the Vertebral-SUV by the Liver-SUV. The ROI for the femoral bone marrow (C) was placed on the caudal side of the lesser trochanter, where the trabecular bone was not visible, and both sides of the mean Hounsfield units (HU) were averaged (BM-CT number).

The BM-CT number was determined as the mean CT attenuation for the bilateral femoral BM (Figure 1). For CT attenuation analysis, femoral bone marrow was selected to minimize contamination from osseous structures. In vertebral bodies, bone marrow is interspersed with abundant trabecular bone, and regions of interest inevitably include high-attenuation trabecular components, which may obscure the true attenuation of marrow tissue. In contrast, the femoral diaphysis contains a relatively large medullary cavity with minimal visible trabecular bone, allowing placement of regions of interest within the marrow space while avoiding both cortical and trabecular bone. Accordingly, femoral bone marrow CT attenuation was considered to better reflect intrinsic marrow attenuation. The BM-CT numbers were measured by two radiologists with 22 and 10 years of experience in CT and nuclear medicine imaging, respectively.

Statistical analysis

Pearson’s correlation coefficients of the BM-CT number were calculated for the BLR, hemoglobin, LDH, serum albumin, CRP levels, NLR, and PLR.

Survival curves were estimated using the Kaplan-Meier method to calculate the recurrence and overall survival (OS) rates. Survival time was defined as the time from the FDG-PET/CT examination to the day of death. Patients without recurrence or death were censored at the date of the last follow-up visit or telephone follow-up survey of electronic medical records. The predictive value of the variables for OS was evaluated using the log-rank test in univariate analysis and the Cox proportional hazards regression model for multivariate analysis. All continuous variables in the survival analysis were dichotomized according to specific cut-off values, which were determined using receiver operating characteristic (ROC) curve analysis. To evaluate the reproducibility of the measurement, the BM-CT number was evaluated using an interclass correlation coefficient (ICC 2,1). The distribution of the data was visually assessed for normality using histograms.

All statistical analyses were performed using SPSS version 20.0 for Windows software (IBM Corp., Armonk, USA), and a P value < .05 was considered statistically significant.

## Results

Patient characteristics

The characteristics of the enrolled patients are summarized in Table 1. Surgery was performed in 105 of 160 patients; 24 people (22.9%) had recurrence, and 22 (21.0%) patients who received surgery died. Of the 55 patients who did not undergo surgery during the clinical follow-up of 60 months, 37 (67.2%) died. The mean interval between preoperative FDG PET/CT imaging and surgical resection or drug, radiation therapy was 35.0 ± 19.7 days. The mean interval between preoperative FDG PET/CT imaging and blood testing was 6.6 ± 4.0 days. The average follow-up period was 44.0 ± 20.3 months.

**Table 1:** Patient characteristics Abbreviations: BLR, bone marrow-to-liver ratio; BMI, body mass index; NLR, neutrophil-to-lymphocyte ratio; PLR, platelet-to-lymphocyte ratio; BM-CT, bone marrow CT; LDH, lactate dehydrogenase; CRP, C-reactive protein

Characteristics	Surgery	No surgery
Age, mean ± SD	70.0 ± 8.3	69.2 ± 10.7
Sex (male/female)	72/33	42/13
Histopathology		
Adenocarcinoma	74	40
Squamous cell carcinoma	21	14
Adenosquamous carcinoma	2	0
Large cell carcinoma	2	0
Adenocarcinoma + squamous cell carcinoma	2	0
Adenocarcinoma + large cell carcinoma	1	0
Polymorphic carcinoma	2	0
Poorly differentiated carcinoma	1	0
Sarcomatoid carcinoma	0	1
Stage		
Stage 0	5	0
Stage I	56	3
Stage II	24	4
Stage III	19	8
Stage IV	1	40
Recurrence (yes/no)	24/81	-
Hemoglobin (g/dL)	13.8 ± 1.3	12.9 ± 1.9
NLR	2.46 ± 1.37	4.30 ± 3.14
PLR	142.9 ± 68.7	218.5 ± 115.6
Albumin (g/dL)	4.24 ± 0.38	3.74 ± 0.58
CRP (mg/dL)	0.25 ± 0.74	2.00 ± 3.70
LDH (U/L)	196.0 ± 43.7	234.0 ± 140.4
BMI	22.7 ± 3.0	22.0 ± 3.30
BLR	0.64 ± 0.14	0.73 ± 0.20
BM-CT number	−38.1 ± 15.4	−23.4 ± 24.9

Relationship between bone marrow CT number and BLR and serum inflammatory markers

Table 2 shows the relationships among the BM-CT number, BLR, and blood biochemistry test results (hemoglobin, LDH, albumin, CRP levels, NLR, and PLR). BM-CT number showed a weak correlation with BLR (γ = 0.257) and BMI (γ = 0.323), In contrast, no significant correlation was observed in BM-CT number between NLR (γ = 0.092), PLR (γ = 0.114), CRP (γ = 0.017), hemoglobin (Hgb) (γ = -0.019), LDH (γ = 0.199), albumin (γ = -0.028) and age (γ = -0.065).

**Table 2:** Relationship between bone marrow CT number and BLR, blood test results, age, and BMI Abbreviations: BLR, bone marrow-to-liver ratio; BMI, body mass index; NLR, neutrophil-to-lymphocyte ratio; PLR, platelet-to-lymphocyte ratio; CRP, C-reactive protein; LDH, lactate dehydrogenase; BM-CT, bone marrow CT

Variables	BLR	Albumin	CRP	NLR	PLR	Hemoglobin	BMI	LDH	Age
BM-CT number	r = 0.257	r = -0.028	r = 0.017	r = 0.092	r = 0.114	r = -0.019	r =0.323	r = 0.199	r = -0.065
	P < 0.001	P = 0.725	P = 0.832	P = 0.247	P = 0.152	P = 0.809	P < 0.001	P = 0.012	P = 0.417
BLR	-	r = -0.241	r = 0.371	r = 0.258	r = 0.118	r = -0.085	r = 0.081	r = 0.083	r = -0.198
		P = 0.002	P < 0.001	P < 0.001	P = 0.118	P = 0.286	P = 0.307	P = 0.299	P = 0.012

Prognostic factors for predicting OS

Age, sex, stage, serum albumin level, CRP level, NLR, PLR, BLR, and BM-CT number were evaluated in the OS analysis. The optimal cutoff values determined using the receiver operating characteristic (ROC) curve analysis were 73 years for age, 4.0 g/dL for serum albumin level, 0.28 mg/dL for serum CRP level, 2.9 for NLR, 205.0 for PLR, 0.665 for BLR, and -32 HU for BM-CT number.

**Figure 2:**
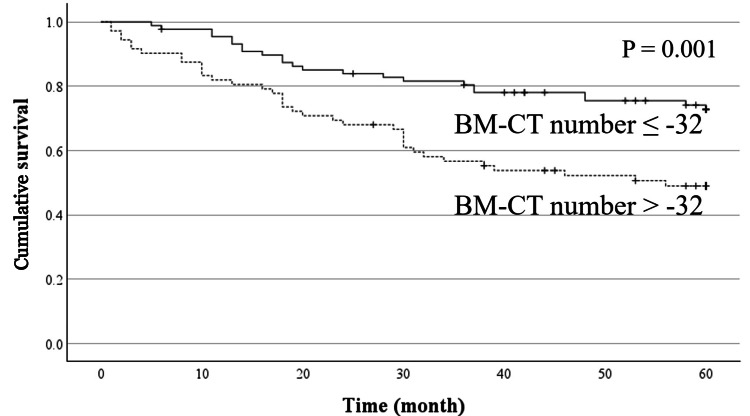
Cumulative overall survival curves based on the bone marrow CT number in all patients Patients with bone marrow CT (BM-CT) numbers > -32 Hounsfield units (HU) showed worse overall survival than those with bone marrow CT numbers ≤ -32.

In analysis of all patients, age (P = 0.006), TNM stage (P < 0.001), hemoglobin (P = 0.009), albumin (P < 0.001), serum CRP level (P < 0.001), LDH (P = 0.003), NLR (P < 0.001), PLR (P = 0.001), and BM-CT number (P = 0.001) were significantly associated with the prognosis in the univariate prognostic analysis using the log rank test, whereas sex (P = 0.065), BMI (P = 0.121), and BLR (P = 0.213) were not (Figure 2, Table 3). Multivariate analysis revealed that age (P = 0.002), TNM stage (P < 0.001), albumin level (P =0.024), and LDH level (P <0.001) were significant independent prognostic factors for OS, but the number of BM-CTs was not (P = 0.218) (Table 3).

**Table 3:** Predictive value of prognostic factors for overall survival in all patients *Median not reached Abbreviations: BLR, bone marrow-to-liver ratio; BMI, body mass index; NLR, neutrophil-to-lymphocyte ratio; PLR, platelet-to-lymphocyte ratio; CRP, C-reactive protein; LDH, lactate dehydrogenase; BM-CT, bone marrow CT; TNM: tumor-node-mestastasis; HU, Hounsfield units

Variables	median survival (month)	P (Univariable analysis)	P (Multivariable analysis)	Hazard ratio	95%CI
Age					
73 ≤	58	0.006	0.002	1.053	1.018-1.089
73 >	*				
Sex					
Male	*	0.065	NA	NA	NA
Female	*				
TNM stage					
≤ Ⅰ	*	< 0.001	<0 .001	4.654	2.101-10.308
> Ⅰ	48				
BMI (body mass index)					
20.8 ≤	*	0.121	NA	NA	NA
20.8 >	*				
Hemoglobin					
14.0 ≤	*	0.009	NA	NA	NA
14.0 >	*				
Albumin					
4.0 ≤	*	< 0.001	0.024	0.481	0.255 – 0.907
4.0 >	32				
CRP					
0.28 ≤	30	< 0.001	0.7	NA	NA
0.28 >	*				
LDH					
238 ≤	39	0.003	< 0.001	1.004	1.002-1.006
238 >	*				
NLR					
2.9 ≤	37	< 0.001	0.970	NA	NA
2.9 >	*				
PLR					
205 ≤	31	0.001	0.168	NA	NA
205 >	*				
BM-CT number					
-32HU≤	56	0.001	0.218	NA	NA
-32HU>	*				
BLR					
0.665 ≤	*	0.213	NA	NA	NA
0.665 >	*				

**Figure 3:**
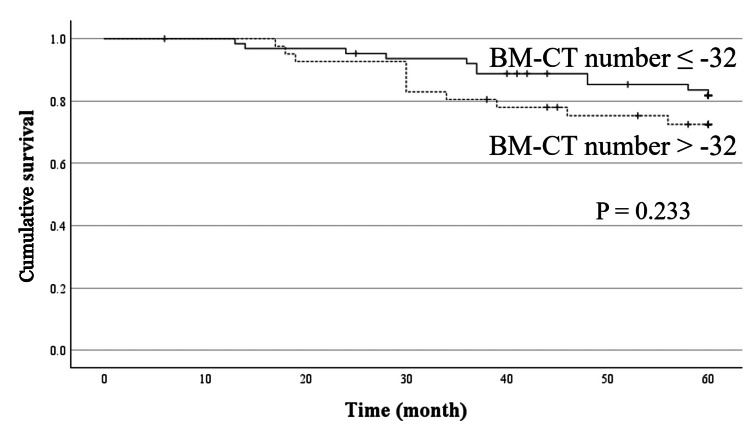
Cumulative overall survival curves based on the bone marrow CT number in patients who had received curative surgery Patients with bone marrow CT (BM-CT) numbers > -32 Hounsfield units (HU) showed worse overall survival than those with bone marrow CT numbers ≤ -32 HU; however, no significant difference was observed between the two groups.

In the analysis of the patients who received curative surgery for NSCLC, age (P < 0.001), TNM stage (P < 0.001), albumin (P = 0.029), serum CRP level (P = 0.020), NLR (P = 0.013), and PLR (P = 0.001) were significantly associated with the prognosis in the univariate prognostic analysis; however, the BM-CT number (P = 0.233) was not (Figure 3, Table 4). Multivariate analysis revealed that TNM stage (P < 0.001) and age (p = 0.01) were significant independent prognostic factors for OS (Table 4).

**Table 4:** Predictive value of prognostic factors for overall survival in patients who received curative surgery for non-small-cell lung cancer. *Median not reached Abbreviations: BLR, bone marrow-to-liver ratio; BMI, body mass index; NLR, neutrophil-to-lymphocyte ratio; PLR, platelet-to-lymphocyte ratio; CRP, C-reactive protein; LDH, lactate dehydrogenase; BM-CT, bone marrow CT; TNM: tumor node mestastasis; HU, Hounsfield units

Variables	Median survival (month)	P (Univariable analysis)	P (Multivariable analysis)	Hazard ratio	95% CI
Age					
73 ≤	*	< 0.001	0.01	1.122	1.029 – 1.224
73 >	*				
Sex					
Male	54.1	0.360	0.215	NA	NA
Female	55.8				
TNM stage					
≤ Ⅰ	*	< 0.01	< 0.001	6.861	2.188 -21.512
> Ⅰ	*				
BMI					
20.8 ≤	*	0.056	NA	NA	NA
20.8 >	*				
Hemoglobin					
14.0 ≤	*	0.222	NA	NA	NA
14.0 >	*				
Albumin					
4.0 ≤	*	0.029	0.617	NA	NA
4.0 >	*				
CRP					
0.28 ≤	*	0.020	0.125	NA	NA
0.28 >	*				
LDH					
238 ≤	*	0.173	NA	NA	NA
238 >	*				
NLR					
2.9 ≤	*	0.013	0.812	NA	NA
2.9 >	*				
PLR					
205 ≤	*	0.001	0.10	NA	NA
205 >	*				
BM-CT number					
-32HU≤	*	0.233	NA	NA	NA
-32HU>	*				
BLR					
0.665 ≤	*	0.862	NA	NA	NA
0.665 >	*				

**Figure 4:**
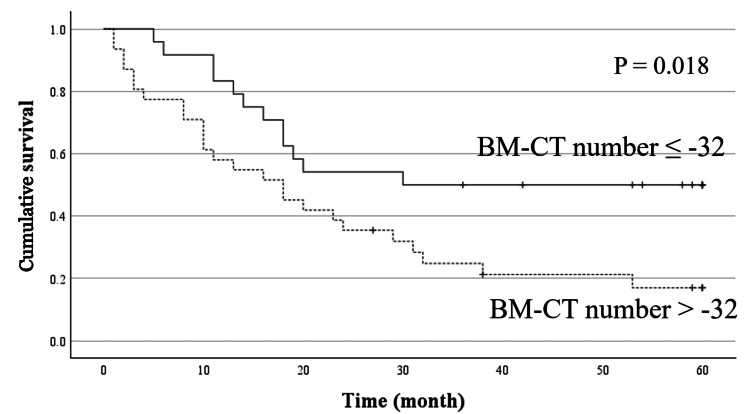
Cumulative overall survival curves based on the bone marrow CT number in patients who had not received curative surgery Patients with bone marrow CT (BM-CT) numbers > -32 Hounsfield units (HU) showed worse overall survival than those with a bone marrow CT number ≤ -32 HU.

In the analysis of the patients who did not undergo curative surgery, the BM-CT number was significantly associated with prognosis in the univariate prognostic analysis (P =0.018) but not in the multivariate analysis (P = 0.123) (Figure 4, Table 5).

**Table 5:** Predictive value of prognostic factors for overall survival in patients who have not received curative surgery Abbreviations: BLR, bone marrow-to-liver ratio; BMI, body mass index; NLR, neutrophil-to-lymphocyte ratio; PLR, platelet-to-lymphocyte ratio; CRP, C-reactive protein; LDH, lactate dehydrogenase; BM-CT, bone marrow CT; TNM: tumor node mestastasis; HU, Hounsfield units

Variables	Median survival (month)	P (Univariable analysis)	P (Multivariable analysis)	Hazard ratio	95% CI
Age					
73 ≤	25	0.254	0.186	NA	NA
73 >	32				
Sex					
Male	19	0.121	0.188	NA	NA
Female	53				
TNM stage					
> Ⅱ	18	0.463	NA	NA	NA
≤ Ⅱ	24				
BMI					
20.8 ≤	23	0.345	NA	NA	NA
20.8 >	16				
Hemoglobin					
14.0 ≤	53	0.105	NA	NA	NA
14.0 >	18				
Albumin					
4.0 ≤	38	0.120	NA	NA	NA
4.0 >	18				
CRP					
0.28 ≤	19	0.923	NA	NA	NA
0.28 >	24				
LDH					
238 ≤	18	0.159	NA	NA	NA
238 >	24				
NLR					
2.9 ≤	18	0.558	NA	NA	NA
2.9 >	23				
PLR					
205 ≤	24	0.907	NA	NA	NA
205 >	20				
BM-CT number					
-32HU≤	25	0.018	0.123	NA	NA
-32HU>	37				
BLR					
0.665 ≤	24	0.151	NA	NA	NA
0.665 >	33				

The ICC (2,1) for the BM-CT number between the two readers was 0.823.

## Discussion

Fluorodeoxyglucose accumulation in the bone marrow reflects its activity in response to inflammation [11, 15, 16]. Furthermore, bone marrow FDG uptake is elevated in patients with lung cancer, which has been reported as a systemic inflammatory response to malignancy [17]. In addition to FDG-based parameters, bone marrow attenuation on CT has also been suggested to reflect marrow cellularity and inflammatory status [11]. Inflammation plays an important role in tumor development and progression. Inflammation promotes tumor cell proliferation and migration, induces angiogenesis, and suppresses antitumor immune responses. Therefore, a state of high inflammation is associated with poor prognosis [18-20].

In this study, the BM-CT number was associated with the prognosis of NSCLC in univariate analysis in patients who did not undergo curative surgery; however, it did not remain significant after adjusting for other covariates in the multivariate model, suggesting the influence of confounding factors. When compared with inflammatory markers, the BM-CT number showed no correlation with NLR (r = 0.092) and CRP level (r = 0.017); however, BLR showed a weak correlation with NLR (r = 0.258) and CRP level (r = 0.371), which is consistent with previous studies [8]. It has been reported that the BM-CT number is negatively correlated with age. Additionally, a BM-CT number exceeding 0 HU is associated with an increased white blood cell count [11]. Furthermore, the number of BM-CTs has been reported to increase in blood disorders and anemia [11, 13].

Bone marrow is typically fat density in healthy adults, and it is presumed that bone marrow density increases owing to cellular infiltration in cases of inflammation. In hematological disorders, elevated bone marrow density may be caused by cellular infiltration or fibrosis. In daily clinical practice, patients with generalized edema may exhibit elevated bone marrow density due to bone marrow edema. Therefore, the interpretation of the BM-CT number should consider potential confounding factors unrelated to tumor biology.

Bone marrow attenuation may be influenced by anemia, hematologic disorders, systemic infection, medication use such as corticosteroids or hematopoietic growth factors, and bone marrow edema associated with poor general condition. These factors may partially explain the attenuation values observed in this study and could not be fully controlled in a retrospective design. Although this association did not persist after multivariate adjustment, the observed univariate relationship between BM-CT number and prognosis in NSCLC patients who did not undergo curative surgery suggests that BM-CT number may reflect underlying systemic conditions related to prognosis rather than serving as an independent prognostic factor. Therefore, the BM-CT number may be interpreted as a surrogate marker reflecting host systemic inflammatory status, providing complementary information for risk stratification rather than functioning as an independent prognostic biomarker.

In parallel with CT-based marrow assessment, FDG uptake-based parameters were also evaluated to contextualize the biological relevance of BM-CT number. Specifically, BLR was used as an indicator of FDG accumulation in the bone marrow. This is because the BM SUV, based on mean 18F-FDG uptake in the liver, reduces the inter-individual variation in the BM SUV [15]. BLR has higher correlation coefficients with serum inflammatory markers than does BM SUV, and it is a preferable parameter for survival analysis [9, 10, 21-23]. However, BLR was not correlated with prognosis, unlike previous studies. The cause is unclear, but possible factors include differences in BLR measurement methods, relatively early-stage cancer in the study cohort, differences in the study population, and the influence of advances in lung cancer treatment on prognosis [2].

Furthermore, owing to recent advances in immune checkpoint inhibitors and molecular-targeted drugs, the prognosis of cancer, including NSCLC, has improved, and the impact of cancer-induced inflammatory activity on prognosis may be weakened compared to that in a previous study [24]. These discrepancies further underscore the complexity of inflammation-related imaging biomarkers and support the interpretation that BM-CT number provides overlapping, but not independent, biological information compared with established PET-based metrics.

This study has several limitations. First, its retrospective single-center design may limit the generalizability of the findings. Second, the study population was heterogeneous with respect to disease stage and treatment strategy, including a substantial proportion of early-stage lung cancers and patients who underwent curative surgery. This heterogeneity may have complicated survival interpretation and potentially attenuated the prognostic impact of imaging biomarkers, including BM-CT number. Nevertheless, such heterogeneity reflects real-world clinical practice and may support the pragmatic relevance of evaluating imaging biomarkers derived from routinely acquired CT data. Prospective multicenter studies with more homogeneous patient cohorts are warranted to validate the prognostic value of the BM-CT number.

Third, although blood biomarkers were ideally expected to be obtained on the same day as the PET/CT examination, laboratory data collected within 14 days of imaging were used in this study, which may have introduced selection bias. Fourth, although interobserver agreement for BM-CT number measurements was acceptable as assessed by the intraclass correlation coefficient, CT attenuation measurements remain subject to inherent variability related to region-of-interest placement, slice selection, and partial volume effects. Even in the femoral diaphysis, subtle inclusion of cortical bone, marrow heterogeneity, or differences in patient positioning may influence measured attenuation values. Therefore, the precision and accuracy of BM-CT measurements should be interpreted with caution, and further standardization of measurement protocols is warranted.

In addition, although the use of a single scanner and reconstruction protocol reduced variability, CT attenuation values remain sensitive to scanner-specific factors and reconstruction kernels, which should be carefully considered in future multicenter studies. Fifth, external validation using independent cohorts was not performed, and therefore, the robustness and generalizability of the proposed cut-off values and prognostic findings require further confirmation. Sixth, this study evaluated the BM-CT number at a single time point, and longitudinal changes in bone marrow attenuation in relation to treatment, disease progression, or systemic inflammation were not assessed. Future studies incorporating serial imaging may help clarify the temporal dynamics and biological significance of marrow attenuation changes.

Finally, the dichotomization of continuous variables based on ROC-derived cut-off values may have reduced statistical power and limited the assessment of dose-response relationships. In addition, the loss of significance of the BM-CT number in multivariate analysis suggests potential confounding or collinearity with established clinical and inflammatory variables.

## Conclusions

The BM-CT number was associated with overall survival in univariate analysis among patients with non-small cell lung cancer who did not undergo curative surgery; however, this association did not remain significant after multivariate adjustment. Therefore, the BM-CT number should be regarded as a hypothesis-generating and complementary imaging marker, rather than an independent prognostic predictor. At present, its clinical utility remains exploratory, and the findings should be interpreted with caution. Nevertheless, given its simplicity and availability from routine CT data, the BM-CT number may serve as a potential adjunct to established clinical and laboratory factors in future prognostic models, pending validation in prospective, multicenter studies.
